# Morphologic and morphometric characteristics of ascaroid worm, *Ophidascaris piscatori* in *Xenochrophis piscator* snake in Sidoarjo, Indonesia

**DOI:** 10.14202/vetworld.2018.1159-1163

**Published:** 2018-08-25

**Authors:** Lucia Tri Suwanti, Inggarsetya Syah Audini, Setiawan Koesdarto, Emmanuel Djoko Poetranto

**Affiliations:** 1 Department of Veterinary Parasitology, Faculty of Veterinary Medicine, Universitas Airlangga, Jl. Mulyorejo, Kampus C Unair, Surabaya, 60115, Indonesia; 2 Master Program Student, Faculty of Veterinary Medicine, Universitas Airlangga, Jl. Mulyorejo, Kampus C Unair, Surabaya, 60115, Indonesia; 3 Department of Veterinary Clinic, Faculty of Veterinary Medicine, Universitas Airlangga, Jl. Mulyorejo, Kampus C Unair, Surabaya, 60115, Indonesia

**Keywords:** carmine, *Ophidascaris piscatori*, scanning electron microscopy, Sidoarjo Indonesia, *Xenochrophis piscator* snake

## Abstract

**Aim:**

The study was conducted to describe the morphology and morphometry of nematode worm in the stomach of *Xenochrophis piscator* snake macroscopically and microscopically using light and scanning electron microscopy (SEM).

**Materials and Methods:**

The sample was 40 nematode worms that have been collected from 60 snakes which slaughtered at snake slaughterhouses in Sidoarjo, Indonesia. The worms (10 male and 10 female) were stained with carmine, and the others were sent to Indonesian Science Institute for ultrastructure observation by SEM. Some of the female worms were immersed in physiological NaCl and incubated to collect the worm eggs.

**Results:**

Nematode worm in this study had three lips with almost the same size and it had papillae, so it was included in ascaridoid. The mouth of ascaridoids has three lips, the dorsal bearing two large outer papillae and the each lateroventral with one papilla. The body length and width of the male worm were 70-105 mm and 0.92-1.32, respectively, with head diameter of 0.22-0.28 mm. Dorsal and ventrolateral lips almost have the same size that was 0.10-0.12×0.11-0.13 mm. The length of interlabia was 0.06-0.08 mm, esophagus was 3.21-4 mm, tail was 0.17-0.23 mm, and spicule was 2.12-3.36 mm. The body length and width of the female worm were 85-130 mm and 1.28-1.71 mm, respectively. The head diameter was 0.29-0.38 mm. Dorsal and ventrolateral lips almost have the same size that was 0.13-0.16×0.15-0.19 mm. The interlabial length was 0.08-0.10 mm, esophagus was 3.04-4.67 mm, and tail was 0.22-0.31 mm. The distance of the vulva from the anterior edge was 56-88 mm with an average of 67.35 mm. The eggs have conspicuously pitted with length 0.08-0.09 mm and width 0.07-0.08 mm.

**Conclusion:**

Based on the characteristics of morphology and morphometry, the ascaroid worms found on *X. piscator* snake from Sidoarjo, Indonesia, were *O. piscatori*.

## Introduction

The *Xenochrophis piscator* is a non-venomous snake that is widely found in Indonesia and distributed on the islands of Sumatra, Java, and Kalimantan [1]. This snake is widely traded and used as traditional medicine (meat, blood, and bile) or accessories (leather) or as a pet [2]. *X. piscator* is also called checkered keelback snake or Asiatic water snake because the habitat of this snake is freshwater lake or river [3].

The most common health problem faced by *X. piscator* snake is an endoparasite infection. According to Rajesh *et al*. [4], reptiles may contain various worms which, according to Klingenberg [5], may cause gastrointestinal obstruction, nutritional deficiencies, and tissue necrosis and are susceptible to secondary infection by bacteria. This will cause the snake to be more susceptible to diseases, affect the body’s appearance (esthetics), and may even cause death.

Various types of worms are reported to infect snakes. One of them that can infect the snake is ascaroid nematode including *Ophidascaris* spp. *Ophidascaris* spp. is commonly found as parasite in snakes worldwide [6], and several researchers have found *Ophidascaris* spp. in various snacks [6-13].

In Tulangan district, Sidoarjo, East Java, Indonesia, there is snake slaughterhouse. The snake products were exported as snake meat and leather. *X. piscator* was one type of snake that is routinely slaughtered, and our previous study [14] found 426 nematode worms from snake’s stomach. This study was conducted to describe the morphology and morphometry of nematode found in the stomach of *X*. *piscator* macroscopic and microscopically using light and scanning electron microscopy (SEM).

## Materials and Methods

### Ethical approval

This study was reviewed and approved by the Animal Care and Use Committee of Faculty of Veterinary Medicine, Universitas Airlangga, number 678-KE.

### Research materials

The research material for routine microscopic examination consisted of aquadest, phosphate buffer saline with pH 7.2, 70%, 85%, and 95% alcohol, 10% formalin, 5% glycerine alcohol, Hung’s I, Hung’s II, and carmine solution, acidic alcohol, alkaline alcohol, and HCl solution.

For SEM examination, the research materials were NaCl solution, buffer phosphate with pH 7.4 for cleaning/washing samples, 2% glutaraldehyde and 1% osmic acid solution for worm fixation, 30%, 50%, 70%, 80%, 90% and absolute alcohols for worm dehydration, and absolute amyl acetate to preserve the worms before dried as well as pure gold or carbon to coat the worms.

### Research tools

The tools used in this study are surgical instruments (such as anatomical tweezers, scalpel blades, and scissors), Petri dishes, plastic trays, microscopic slides, cover glass (coverslip), scissors, beaker glass, Erlenmeyer, staining jar, disposable syringe, tissue or paper towels, tube rack, plastic pipette, Glass Graduated Cylinder, light microscope, and JSM-5000 SEM.

### The sample

The samples were 40 nematode worms (20 male and 20 female) that have been collected from the stomach of 60 *X. piscator* snakes that slaughtered in district Tulangan, Sidoarjo, East Java, from December 2016 to April 2017. The worms taken from the stomach of the snake were washed and immersed in a NaCl 0.9% solution and were stored and identified at the Department of Veterinary Parasitology, Faculty of Veterinary Medicine, Universitas Airlangga.

### Macroscopic identification

Macroscopic identification was done by observing the color, anterior and posterior shapes, and body length of the worm.

### Microscopic identification

Twenty (10 male and 10 female) worms were stained with carmine. Worms were immersed in a 5% glyceride alcoholic solution for 24 h and then in 70% alcohol for 5 min. The worms were transferred into an alcoholic carmine solution (1: 2) for approximately 8 h, and then, it was soaked into acidic alcohol for 10 min and alkaline alcohol for 20 min. Respectively, the worms were dehydrated into 70%, 85%, and 95% alcohol for 5 min. Mounting was done with Hung’s I solution for 20 min and placed on clean object glass. Hung’s II solution was dripped sufficiently over the worm and covered with a cover glass. Slides were dried in the incubator at 37°C [15]. Worms were observed using a light microscope based on the method of Soota and Chaturvedi [7] with 40 and 100×.

To get worm eggs, some female worms were incubated in buffer saline at 37°C for 24 h. The day after, buffer saline was centrifuged at 1500 rpm for 5 min, and sediment was dropped in slide glass and was observed under a light microscope with 100×. Twenty nematode worms were sent to the Indonesian Science Institute for ultrastructure observation by SEM.

## Results

**Figure-1:**
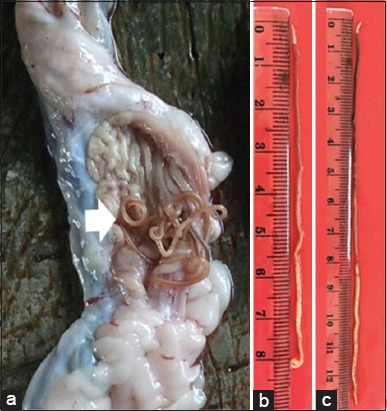
Fresh ascaroid nematode worms. (a) Worms in the stomach of *Xenochrophis piscatori* (white arrow). (b) Male worm. (c) Female worm.

The nematode worms were found inside the snake stomach (Figure-1a). Macroscopically, the worm appears brownish on the anterior and white on the posterior. The posterior body part is wider than the anterior portion, especially in the female worm (Figure-1b and c). The female worms have a larger size and length than the male (Figure-1c).

**Figure-2:**
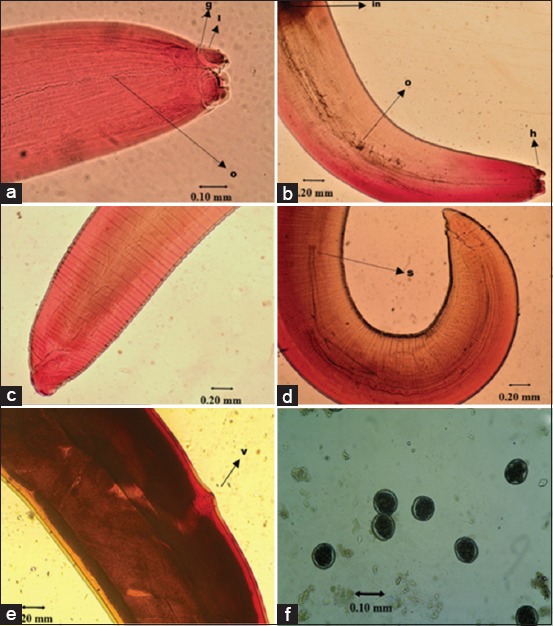
*Ophidascaris piscatori* with carmine stain. (a and b) anterior (×100 and ×40). (c and e) Posterior of female worm (×40). (d) Posterior of male worm (×40). (f) Worm eggs (×100). g: Post-labial groove; l: Lips; o: Esophagus; in: Intestine; s: Spicule, v: Vulva.

**Figure-3:**
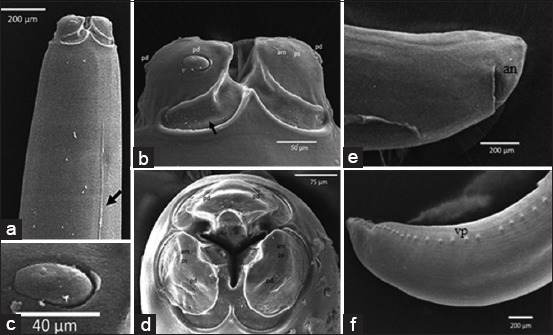
*Ophidascaris piscatori* with scanning electron microscopy. (a-d) Anterior; (e and f) posterior; (a) ventrolateral with cervical alae (black arrow). (b) The head with lips, interlabial, and deep post-labial grooves. (c) Detail of double papillae. (d) Apical of the head; (e) Posterior of female worm; (f) posterior of male worm. am: Amphid; an: Anus; pd: Double papillae; ps: Single papillae, vp: Ventrolateral papillae.

The shape of the mouth is similar to the other ascaridoid, which has three lips with almost the same size (Figures-2a, 3a, 3b and 3d). The dorsal portion of the lips has two double papillae, whereas in the lateroventral lips, each has one double papilla, one single papilla, and an amphid (Figures-3b and 3c). There was interlabial, which is one of the characteristics of ascaroid worm. Interlabia develops well with a deep post-labial groove (Figure-3b), with an interlabial length of nearly half the size of the lip length (Figure-3d). Ventrolateral anterior has *cervical alae* (Figure-3a). The esophagus and the border between the esophagus and intestine are clearly visible (Figure-2b). The cuticles are appeared with transverse striation (Figure-2b-d). The posterior portion of the male worm is bent to ventral, and there are spicules that are sometimes protruded (Figures-1b and 2d), and in ventrolateral, there is a row of papillae (Figure-3f). In the posterior portion of the female worm, the posterior edges are rounded, while in the male worm, the posterior end is tapered (Figure-1c and 2d). The female vulva of the female worm lies approximately two-third from the anterior body (Figure-2e). The anus is located near the posterior end (Figure-3e).

The body length and width of the male worm were 70-105 mm (87.2 mm) and 0.92-1.32 (1.13 mm), respectively. The head diameter was 0.22-0.28 mm (0.26 mm). Dorsal and ventrolateral lips almost have the same size that was 0.10-0.12×0.11-0.13 mm (0.10×0.12 mm). The length of interlabia was 0.06-0.08 mm (0.07 mm), esophagus was 3.21-4 mm (3.58 mm), tail was 0.17-0.23 mm (0.19 mm), and spicule was 2.12-3.36 mm (2.88 mm) with rounded edges.

The body length and width of the female worm were 85-130 mm (103 mm) and 1.28-1.71 mm (1.46 mm). The head diameter was 0.29-0.38 mm (0.33 mm). Dorsal and ventrolateral lips almost have the same size that was 0.13-0.16×0.15-0.19 mm (0.14×0.18 mm). The interlabial length was 0.08-0.10 mm (0.09 mm), esophagus was 3.04-4.67 mm (3.8 mm), and tail was 0.22-0.31 mm (0.26 mm). The distance of the vulva from the anterior edge was 56-88 mm with an average of 67.35 mm. The egg length and width were 0.08-0.09 mm and 0.07-0.08 mm. The egg was rounded with a shell that has conspicuous pits (Figure-2f).

## Discussion

Nematode worm in this study had three lips with almost the same size and it had papillae, so it was included in ascaridoid. The mouth of ascaridoids has three lips, the dorsal bearing two large outer papillae and the each lateroventral with one papilla [6]. Ascaridoid nematodes are very common in snakes, and *Ophidascaris* is an Ascarididae family of nematode often found in reptiles [12].

Some researchers had identified *Ophidascaris* species. Differential features of lip shape, esophageal length and spicules, tail, the number and arrangement of paracloacal and postcloacal papillae, and egg morphology are commonly used to recognize different species of *Ophidascaris* [9]. *Ophidascaris* *durissus* had found from *Crepitus durissus* snake in Brazil [6], *Ophidascaris filaria* was found in an Indian python in Sistan [10], and *Ophidascaris* *excavata* was found in the pit viper *Gloydius brevicaudus* in the Republic of Korea [11].

Based on host and morphologic and morphometrics of worm, compared with the method of Soota and Chaturvedi [7], the ascaridoid nematode found in this study was *O. piscatori*, although there were some difference sizes, especially on lip width which is more narrow as found in this study (Table-1) [7].

**Table-1:** Morphology and Morphometry*Ophidascaris*piscatori.

Characteristic	*Ophidascarispiscatori*		*Ophidascarispiscatori* [7]	
	
	Male (mm)	Female (mm)	Male (mm)	Female (mm)
Body length	70-105 (87.2)	85-130 (103)	85.17-86.54	86.16-95.3
Maximal Width	0.92-1.32 (1.13)	1.28-1.71 (1.46)	1.38-1.4	1.51-1.54
Diameter of Head	0.22-0.28 (0.26)	0.29-0.38 (0.33)	0.28-0.29	0.31-0.33
Lips (length×width)	0.10-0.12×0.11-0.13 (0.10×0.12)	0.13-0.16×0.15-0.19 (0.14×0.18)	0.09-90.11×0.11-0.12	0.14-0.17×0.2-30.26
Length of interlabia	0.06-0.08 (0.07)	0.08-0.10 (0.09)	0.07-0.08	0.088-0.11
Length of the esophagus	3.21-4 (3.58)	3.04-4.67 (3.8)	3.58-3.97	3.74-3.85
Length of tail	0.17-0.23 (0.19)	0.22-0.31 (0.26)	0.2-0.21	0.3-0.33
Distance of vulva from the anterior	-	56-88 (67.35)	-	57.23-64.5
Length of spicule	2.12-3.36 (2.88)	-	2.97-3.03	-
Egg size	-	0.08-0.09×0.07-0.08 (0.08×0.076)	-	0.088-0.0990.066-0.077

*O*. *piscatori* has the following characteristics: Predilection in snake stomach, roundworm, whitish color with brownish in anterior portion, the mouth with three lips similar in size, interlabial, deep post-labial grooves, present cervical alae, and a clear border between the esophagus and intestine. Vulva of the female worm lies approximately two-third from the anterior body and posterior part of the male worm is bent ventral, and there were spicules that were sometimes protruded.

In Indonesia, *Ophidascaris* spp. had also found in Python snakes form Bali [13] and Depok and Bogor [16]. They just found eggs of worm, and based on the examination, the size and shape of eggs were different from this study. *Ophidascaris* spp. eggs found in Bali are an oval-shaped and thick shell with length and width average 0.09 and 0.04 mm. In this study, the egg has conspicuously pitted with length and width average 0.08 and 0.076 mm.

## Conclusion

Based on the characteristics of morphology and morphometry, the ascaroid worms found on *X. piscator* snake from Sidoarjo, Indonesia, were *O. piscatori*. Body length of the male worm was 70-105 mm, and the female was 85-130 mm. The eggs have conspicuously pitted with length 0.08-0.09 mm and width 0.07-0.08 mm.

## Authors’ Contributions

LTS, ISA, SK, and EDP designed the concept for this research and scientific paper. LTS and ISA conducted the research, for example, collecting samples and did the laboratory work. All authors participated in draft and revision of the manuscript and approved the final manuscript.
